# Theoretical Studies of [2,3]-Sigmatropic Rearrangements of Allylic Selenoxides and Selenimides

**DOI:** 10.3390/molecules14093229

**Published:** 2009-08-28

**Authors:** Craig A. Bayse, Sonia Antony

**Affiliations:** Department of Chemistry and Biochemistry, Old Dominion University, Hampton Blvd., Norfolk, VA 23529, USA

**Keywords:** [2,3]-sigmatropic rearrangements, selenoxides, selenium, density-functional theory

## Abstract

Density-functional theory is used to model the *endo* and *exo* transition states for [2,3]-sigmatropic rearrangement of allylic aryl-selenoxides and -selenimides. The *endo* transition state is generally preferred for selenoxides if there is no substitution at the 2 position of the allyl group. Based upon the relative energies of the *endo* and *exo* transition states, enantioselectivity of rearrangements is expected to be greatest for molecules with substitutions at the 1- or (E)-3- position of the allyl group. *Ortho* substitution of a nitro group on the ancillary selenoxide phenyl ring reduces the activation barriers, increases the difference between the *endo* and *exo* activation barriers and shifts the equilibrium toward products.

## 1. Introduction

**Figure 1:**
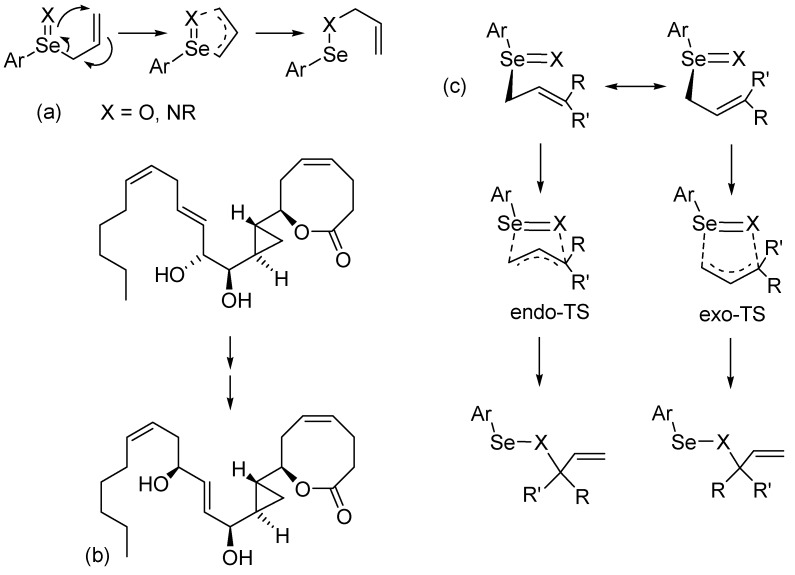
(a) Recent synthetic application of the [2,3]-sigmatropic rearrangement in the total synthesis of solandelactone E^3^. (b) Mechanism of [2,3]-sigmatropic rearrangements (c) Reaction pathways through the endo and exo conformations at the transition state.

[2,3]-Sigmatropic rearrangements of allylic selenoxides and selenimides (Figure 1a) are important tools for the synthesis of primary, secondary and tertiary allylic alcohols and amines [1]. Examples of applications of interest to natural products and bioorganic chemistry include the conversion of A-type prostaglandin to J-type [2], the enantioselective total synthesis of the marine oxylipin solandelactone E [3], as well as in the synthesis of sterols [4] and non-natural amino acids [5]. [2,3]-Sigmatropic rearrangements of selenoxides have also been used for intrastrand cross-linking of DNA [6]. As shown in the synthesis of solandelactone E (Figure 1b), the rearrangement tolerates a variety of functional groups. Enantioselective synthesis of chiral allylic alcohols and amines is possible when either the selenoxide is produced using a chiral oxidizing agent or a chiral ancillary group including those that stabilize the selenoxide against racemization [1,7,8,9,10].

The reaction proceeds by oxidation of an allylic selenide to the selenoxide which undergoes rearrangement to the allylic selenenate. Hydrolysis of the selenenate yields the allylic alcohol. Alternatively, the selenide may be converted to the selenimide by treatment with chloramine T or *N*-chlorosuccinimide and protected amine [11,12,13]. Solvolysis and oxidation of the resulting allylic amine may be used to synthesize amino acids [5]. Reich and coworkers reported the activation barrier of the selenoxide rearrangement of an allylic *o*-nitrophenylselenoxide as approximately 12.5 kcal/mol (ΔG^‡^), with 2 kcal/mol separating distinct transition states where the aryl group is *endo* or *exo* to the allyl group (Figure 1c) [14]. The equilibrium favors the selenenate ester (ΔG = ~ -11 kcal/mol), such that the reaction is effectively irreversible. In contrast, the analogous sulfoxide is slightly more stable than the sulfenate ester (ΔG = 1.5 kcal/mol) with a larger barrier to rearrangement [ΔG^‡^ = 19.8 kcal/mol (*endo* TS)] [14].

In this paper, the transition states for [2,3]-sigmatropic rearrangements of aryl allyl selenoxides and selenimides are modeled using density-functional theory (DFT) and compared to Reich *et al’s* experimental data, as well as previously estimated barriers for the sulfoxide rearrangement [15]. The effect of *ortho* substitution of a nitro group on the activation barriers is also examined as computational studies of selenoxide elimination [16,17] have shown that groups capable of intramolecular Se···N,O interactions [18] substantially lower the barrier to elimination. 

## 2. Results and Discussion

**Figure 2:**
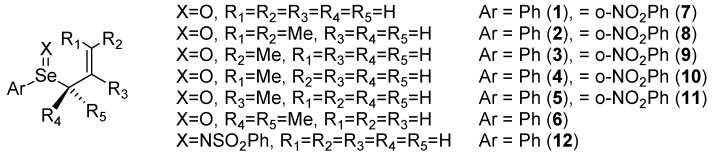
Selenoxides and selenimides under examination in the present study.

**Figure 3:**
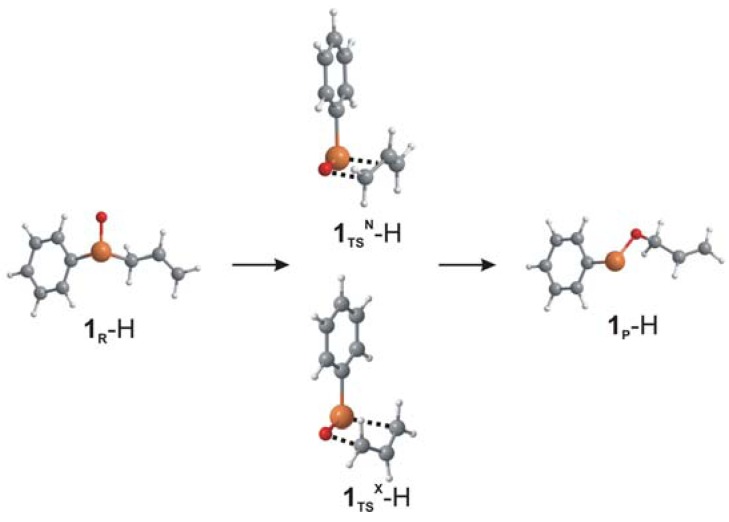
Optimized structures for the selenoxide, *endo* and *exo* transition states and product selenenate for **1**.

**Figure 4:**
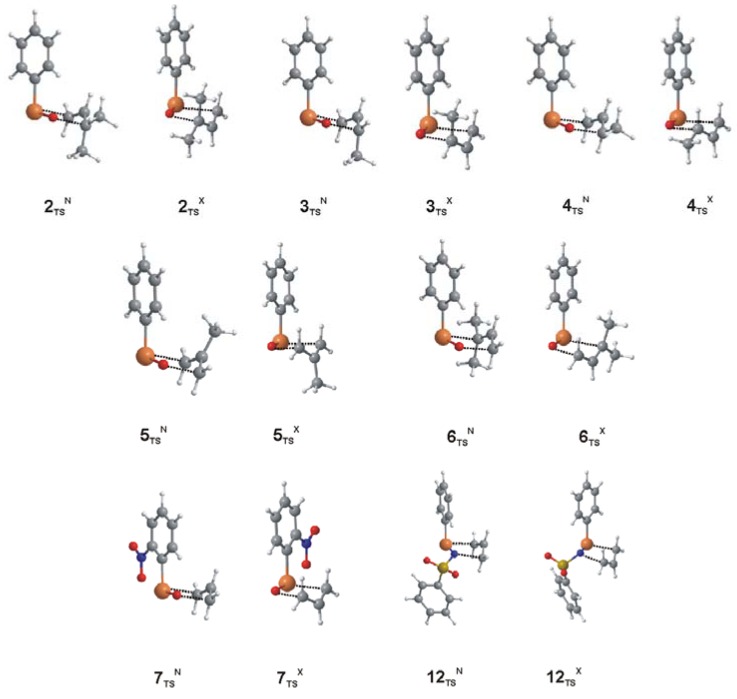
Optimized structures of the endo and exo transition states for **2** through **6**, **7** and **12**.

Calculations were performed on the model allylic phenylselenoxides **1**–**6**, their o-nitrophenyl analogues (**1**–**5** only, *i.e*., **7**–**11**) and the *N*-benzenesulfonimide **12 **(Figure 2) using the B3PW91 exchange correlation functional. Transition states were obtained by a manual scan of the X–C (X=O, NSO_2_Ph) reaction coordinate followed by full optimization to a saddle point. The rearrangement mechanism was assumed to be concerted and both the *endo*- and *exo*- conformations of the transition state were obtained for each model compound. Reaction pathways were examined for a single enantiomer of the selenoxide as if generated from a chiral oxidant. Activation barriers were calculated from the lowest conformation of the reactant selenoxide that leads to these two transition states. The activation barriers and selected geometric parameters for the lowest energy transition states (*endo* or *exo*) for the rearrangement of the selenoxides **1**–**11 **and the selenimide **12** are listed in Table 1 and shown in Figure 3 and Figure 4. In the following discussion, relative energies are calculated from the lowest energy conformation of the selenoxide and discussed in terms of the Gibbs free energy (ΔG) unless otherwise noted. Subscripts are used to designate the selenoxide (R), transition state (TS) and selenenate (P). Superscripts are used to label the transition states as *endo* (N) or *exo* (X).

For the unsubstituted **1**, the energy of the selenoxide (**1_R_**) conformation leading to the *endo* transition state is more stable than the *exo* conformation by 0.4 kcal/mol. Electrostatic interactions between the hydrogen at the 2 position of the allyl group and the phenyl ring stabilizes the *endo* transition state **1_TS_^N^** slightly (0.3 kcal/mol) relative to **1_TS_^X^** (Figure 3). The five-membered transition state **1_TS_^N^** occurs when the O-C distance has shortened to ~2 Å, the Se-O distance has lengthed by 0.04 Å and the Se-C bond being broken has increased by 0.47 Å to 2.48 Å. The bond distances for the *exo* transition state **1_TS_^X^** are similar with a slightly longer C-O bond distance (2.039 Å). The activation Gibbs free energy of 13.2 kcal/mol is in excellent agreement with the experimental ΔG^‡^ determined by Reich *et al*. although the energies of the overall reaction (-7.7 kcal/mol) and the relative energies of the *endo* and *exo* transition states (ΔΔG^‡^_N-X_) were lower due to truncation of the model compound. For comparison, activation barriers for the sulfoxide analogue of **1** (**1**(S)) were calculated. As for **1**, the *endo* conformation of the sulfoxide and the transition state are slightly favored (0.4 kcal/mol). The structures of **1**(S)**_TS_^N^** and **1**(S)**_TS_^X^** and the corresponding activation barriers (16.4 and 16.8 kcal/mol, respectively) are consistent with previous computational results reported by Freeman *et al.* [15] using the B3LYP exchange-correlation functional in conjunction with Pople and correlation-consistent basis sets for allyl sulfoxide models with H, Me, CF_3_ or CCl_3_ instead of an aryl group. Replacement of the selenoxide by a selenimide (**12**) increases the activation barrier for rearrangement slightly to 13.4 kcal/mol, with the *exo* transition state favored by 0.6 kcal/mol. The ΔG for the overall reaction is more negative such that the equilibrium is strongly shifted toward products. The bond distances at the transition state indicate that the transition state is earlier on the reaction pathway with the Se-C bond distance increased by only 0.34 Å and the C-N distance at 2.28 Å. 

*Ortho* substitution of Lewis basic groups such as nitro would be expected to reduce the barrier to [2,3]-sigmatropic shift as a result of the intramolecular Se···O interaction. Donation of electron density from the nitro group to the selenoxide increases the hypervalency of the selenium center favoring single Se-O bond character and increasing the basicity of the selenoxide oxygen [18]. In our examination of selenoxide elimination reactions of Se-substituted selenocysteines [16], the computed activation barrier for *Se*-o-nitrophenylselenocysteine selenoxide was ~3 kcal/mol lower than the para-substituted analogue. For the selenoxide **7_R_**, the Se···O interaction is ~2.75 Å, with little change in the Se=O bond distance relative to **1_R_** (1.66 Å) or the partial charge of the selenoxide oxygen. The *o*-nitro group reduces the energy difference between the conformation of the reactant selenoxide leading to the exo conformation relative to that of the *endo* conformation (ΔΔG_N-X_ < 0.2 kcal/mol), but increases the relative free energies of the activation barriers (ΔΔG^‡^_N-X_) by 0.4 kcal/mol (Table 1) for **7** versus **1**. The transition state **7_TS_^N^** is slightly lower than the unsubstituted selenoxide due to stabilization of the emerging selenenate by the nitro group. The greater exothermicity of the overall rearrangement (ΔH = -15.1 kcal/mol versus -7.7 kcal/mol for **1**) results from a stronger donor-acceptor interaction for the nitro-selenenate pair in comparison to the nitro-selenoxide. The ΔG^‡^ value for the *endo* rearrangement is in good agreement with Reich *et al’s* experimental value. However, for the sulfur analogue **7**(S), the calculated barrier and reaction energy is substantially lower than the experimental values (ΔG^‡^ = 14.2 versus 19.8 kcal/mol and ΔG = -7.3 versus 1.5 kcal/mol). This difference may be attributed to truncation of the model or an underestimation in the DFT method. Freeman *et al.* [15] showed that MP2 barriers are consistently higher than values calculated using B3LYP in the same basis set.

Methyl substitutions on the allyl group vary the activation barriers and the preferred conformation at the transition state. For the overall structures at the transition state (Table 1 and Figure 4), methyl substitution does not affect the Se-O distance, but generally increases the values of the Se-C and C-O distances. Unlike all other compounds in this study, the preferred reactant conformation of the 3,3-dimethylallyl selenoxide **2_R_** and the (Z)-3-methyl substituted **3_R_** are those leading to the *exo* transition state due to favorable electrostatic interactions between the selenoxide and the methyl group. However, for each of these species, the *endo* transition states **2_TS_^N^** and **3_TS_^N^** are lower in energy by 1.2–1.4 kcal/mol due to steric interactions between the *cis*-methyl group and the phenyl ring in the *exo* transition states **2_TS_^X^** and **3_TS_^X^**. For the (E)-3-methylallyl selenoxide **4_R_**, the positioning of the methyl group does not have similar steric interactions and the *endo* conformation is favored by less than 0.1 kcal/mol. Davis and Reddy report that oxidation of (*E*)- and (*Z*)-phenylcinnamyl selenide **9** with chiral oxidizing agents provide some stereoselectivity (40-60% ee) due to the relative activation barriers of the *endo* and *exo* transition states [7]. The computed ΔΔG^‡^_N-X_ value (1.4 kcal/mol) for the *cis* 3-methyl substituted **3** is consistent with both the stereoselectivity and the *endo* transition state. However, Davis and Reddy report that (E)-**9** favors the *exo* transition state with a slightly lower enantiomeric purity (25-40% ee) [7]. The small ΔΔG^‡^_N-X_ difference for the (E)-3-methylallyl transition state **4_TS_** (0.1 kcal/mol) favoring the *endo* conformation may indicate that the steric requirements of different groups at the (E)-3 position may be sufficient to reverse the energy ordering of the transition states. Methyl substitution at the 2-position of the allyl group (**5**) increases the activation barrier and reverses the order of the *endo* and *exo* transition states (ΔΔG^‡^_N-X_ = -1.0 kcal/mol) due to the steric interactions between the methyl group and the phenyl group in the *endo* conformation. For **6**, steric interactions between one of the methyl groups and the phenyl ring are present in both conformations of the transition state, however, **6_TS_^N^** is preferred by 1.3 kcal/mol because the methyl group in **6_TS_^X^** in closer contact with the phenyl ring (see Figure 4). Substitution of a nitro group on the ancillary phenyl group (**7**–**11**) tends to increase ΔΔG^‡^_N-X_ by 0.4–0.7 kcal/mol, potentially leading to greater selectivities in these molecules.

**Table 1:** Selected geometric parameters and activation barriers for the [2,3]-sigmatropic rearrangement of selenoxides **1** – **5**.

	Type ^a^	d(Se/S-O,N), Å	d(Se/S-C), Å	d(C-O,N), Å	ΔG^‡^, kcal/mol	ΔΔG^‡^_N-X_, kcal/mol	ΔG, kcal/mol
1	N	1.699	2.481	2.006	13.2	0.3	-7.7
1(S)	N	1.566	2.463	1.994	16.4	0.4	-1.8
2	N^b^	1.697	2.567	2.077	12.7	1.3	-6.2
3	N^b^	1.696	2.513	2.060	13.5	1.4	-7.4
4	N	1.699	2.521	2.026	12.0	0.1	-7.8
5	X	1.696	2.513	2.049	14.4	-1.0	-7.7
6	N	1.693	2.616	2.122	11.1	1.3	-7.4
7	N	1.695	2.477	2.075	11.2	0.7	-15.1
7(S)	N	1.562	2.462	2.050	14.2	0.9	-7.3
8	N^b^	1.693	2.561	2.157	10.4	1.7	-12.9
9	N^b^	1.692	2.510	2.134	11.4	2.1	-14.1
10	N	1.696	2.517	2.099	9.9	0.4	-14.9
11	X	1.694	2.508	2.102	12.4	-0.9	-15.9
12	X	1.775	2.441	2.276	13.4	-0.6	-19.0

^a^ Conformation of the lowest transition state; ^b^ The lowest reactant conformation of the selenoxide is that leading to the exo TS.

## 3. Theoretical Section

Geometries were optimized with the DFT(B3PW91) exchange-correlation functional using PQS version 3.3 [19]. The Dunning split-valence triple-ζ plus polarization function basis set (TZVP) [20] was used for nitrogen and oxygen. Selenium was represented by the Hurley *et al.* [21] relativistic effective core potential (RECP) double-ζ basis set augmented with a set of even-tempered s, p, and d diffuse functions. Diffuse s- and p-functions were also added to the Wadt-Hay RECP basis set for sulfur [22] and the nitrogen and oxygen atoms. Double-ζ basis sets with polarization functions added to the carbon atoms were used for hydrocarbon fragments [23]. This selection of basis sets has been shown to be effective in our previous studies of organoselenium compounds [16,18,24]. Transition states were determined by manual scan of the reaction pathway followed by full optimization of the preliminary structure. Each of the reported transition state structures was found to have one imaginary mode corresponding to the motion along reaction coordinate. The reported energies include zero-point energy (ZPE), thermal and entropy corrections.

## 4. Conclusions

The above modeling of selenoxide rearrangements generally confirm the experimental results obtained by Reich *et al.* [14]. Additionally, we have shown that *ortho*-substitution of a nitro group (a) lowers the activation barrier, (b) reduces the relative energy between the conformations of the selenoxide leading to the *endo* and *exo* transition states, (c) increases the difference between the *endo* and *exo* transition states ΔΔG^‡^_N-X_, and (d) shifts the equilibrium further toward products. Methyl substitutions at the 1- and (E)-3-positions of the allyl group are expected to provide the greatest enantioselectivity. Substitutions at the 2 position of allyl group reverse the ordering of the transition states due to steric interactions between the substituent and the ancillary phenyl group which favor the *exo* transition state. 
